# 

**Published:** 1855-09

**Authors:** 


### CONTENTS

ORIGINAL COMMUNICATIONS— PAGE.
A case of Eclampsia. By D. W. Stormont, M. D , - - 393
A Case. By G. W. Parkins, M.D., - - - - 398
Pathology of Fevers. By N. S. Davis, M.D., - - - 401
SELECTIONS—
Foreign Correspondence. - - - - - - - - 409
Hæmoptysis as a Sign of Tubercle—Curability of Consumption. 413
The Late Dr. Elisha Bartlett, - - - - - - - 417
Glass Brushes for applying Fluid Caustics, - - - - 421
BOOK NOTICES— 422—425
EDITORIAL— 425—439

